# Sex difference: an important issue to consider in epidemiological and clinical studies dealing with serum paraoxonase-1

**DOI:** 10.3164/jcbn.18-73

**Published:** 2019-01-30

**Authors:** Alessandro Trentini, Tiziana Bellini, Gloria Bonaccorsi, Carlotta Cavicchio, Stefania Hanau, Angelina Passaro, Carlo Cervellati

**Affiliations:** 1 Department of Biomedical and Specialist Surgical Sciences, Section of Medical Biochemistry, Molecular Biology and Genetics, University of Ferrara, Via Luigi Borsari 46, 44121, Ferrara, Italy; 2 University Center for Studies on Gender Medicine, University of Ferrara, Via Luigi Borsari 46, 44121, Ferrara, Italy; 3 Department of Morphology, Surgery and Experimental Medicine, Menopause and Osteoporosis Centre, University of Ferrara, Via Luigi Borsari 46, 44121, Ferrara, Italy; 4 Department of Life Sciences and Biotechnology, University of Ferrara, Via Luigi Borsari 46, 44121, Ferrara, Italy; 5 Department of Medical Sciences, Internal Medicine and CardioRespiratory Section, University of Ferrara, Via Luigi Borsari 46, 44121, Ferrara, Italy

**Keywords:** arylesterase, body mass index, sex difference, waist circumference, obesity, paraoxonase-1

## Abstract

The aim of this study was to evaluate the influence of sex on serum paraoxonase-1 (PON1) activities and on its relationship with cardiovascular disease risk factors such as overall and central obesity. Arylesterase and lactonase activities of PON1 were assessed in 374 women and 92 men. Both arylesterase and lactonase activities were significantly higher in women compared to men (*p*<0.001), irrespectively of confounders such as high density lipoprotein-cholesterol, age, smoking and body mass index or waist circumference. Sex also strongly influenced the interplay between PON1 and both fat measures, with only the arylesterase showing a significant and independent inverse correlation with the former parameter (*r* = −0.248, *p*<0.001) and the risk of overall obesity (odds ratio: 0.559, 95% confidence interval: 0.340–0.919) in women, but not in men; conversely, neither of the two activities remained associated with waist circumference in men or women after full adjustment. Noteworthy, the association between arylesterase and BMI in the female subsample was significant among women younger than forty-five years (*r* = −0.453, *p*<0.001, *R*^2^ = 0.207). In conclusion, our study suggests that sex might chiefly influence PON1 activity and its contribution to cardiovascular disease risk. Further studies are needed to confirm and clarify our preliminary findings.

## Introduction

In the biomedical field, sex refers to the genetic and biological status while gender refers to the social and cultural differences between females and males. As both factors play together, they contribute to the widely reported differences in life expectancy and in the incidence/prevalence of several diseases between women and men.^(1)^ Within this research report, we will employ the term “sex” to include both biological and environmental influence on variables considered.

Cardiovascular diseases (CVDs) represent the field where sex difference has been most extensively explored, with women showing a lower risk of developing these diseases compared to men.^(2)^ This difference is more evident at a young age and significantly decreases after menopause, suggesting a wide protective effect of estrogens (in particular of estradiol, E2).^(3)^ Indeed, the menopause-related decline in E2 is associated with an increased incidence of several disturbances and diseases, including CVDs. One of the most harmful effects of menopause is the change in body fat distribution; estrogen withdrawal promotes visceral/abdominal adiposity shifting fat distribution from gynoid (gluteal-femoral) to android (trunk-central) type.^(4–6)^ In both men and women, central obesity is independently associated with an adverse CVD risk profile with enhanced insulin resistance, increase in LDL-C and triglycerides and decrease in both concentration, size and function of HDL.^(7)^

In line with the above observations, we have recently found that in women overall obesity is significantly, although weakly, associated with a decrease in the activity of one of the most important contributor of HDL athero-protective function, paraoxonase-1 (PON1).^(8)^ This HDL-bound esterase/lactonase enzyme confers antioxidant protection to lipoproteins (HDL and LDL), macrophages and endothelial cells, and appears to co-activate the cholesterol efflux from macrophages.^(9,10)^ PON1 can also exert arylesterase and paraoxonase activities on synthetic chemicals.^(11)^ Noteworthy, paraoxonase and, although to a lesser extent, lactonase activities are influenced by some common single nucleotide polymorphisms (SNPs) of PON1 gene; on the contrary, arylesterase activity is minimally affected by SNPs and, thus, more suitable (lower inter-individual variability) for epidemiological studies.^(12–14)^ Low serum levels of arylesterase activity have been found in association with various diseases including CVD, dementia-related disorders, multiple sclerosis and metabolic syndrome.^(15–18)^ In general, alteration in PON1 activity appears to be implicated in those pathological conditions where the axis oxidative stress-inflammation plays a pathogenic role.^(19)^ Despite the large body of epidemiological evidence that have been collected, the causal role of PON1 in these diseases is still uncertain. The incomplete knowledge of the biological/physiological factors that can modulate PON1 function could be one reason underlying this failure. In this study, we focused on the potential influence of sex on arylesterase/lactonase serum activities of PON1 and its impact on the relationship between these activities and body fat mass.

In this light, the present study sought to evaluate whether and to which extent sex influences PON1 activity and its association with well-established CVD risk factors such as overall and abdominal body fat. To address this aim, we examined the levels of arylesterase/lactonase activities in relation to body mass index (BMI) and waist circumference in a large population-sample of *n* = 374 women and *n* = 92 men.

## Materials and Methods

### Subjects

The present study consisted in the re-examination of data collected from three different cohorts: 1) subjects attending the metabolic outpatient clinic of Sant’Anna University Hospital (Ferrara, Italy); 2) outpatients undergoing bone densitometry testing at the Menopause and Osteoporosis Centre of the University of Ferrara; 3) outpatients referring to the Obesity Centre of Sant’Anna (Ferrara, Italy).

The research protocols conform to The Code of Ethics of the World Medical Association (Declaration of Helsinki) and were conducted accordingly to the guidelines for Good Clinical Practice (European Medicines Agency). The studies were approved by the Local Ethics Committee of the University of Ferrara, and written informed consent was obtained from each patient during the first office visit before the possible inclusion in the study. No personal information was available to the authors of the study in order to protect the anonymity of the patients.

Clinical and laboratory data were collected from each patient at admission, with a complete medical history and physical examination. Only Caucasian men/women older than 18 years were considered as eligible in the study. Subjects affected by chronic diseases, such as diabetes, cancer, malabsorption, dementia, and CVD or by current illness were excluded. Hypothyroidism, pregnancy, alcohol consumption >10 g daily, active treatment with hormones or lipid-modifying drugs were considered exclusion criteria. Other details on inclusion/exclusion criteria have been reported previously.^(20–22)^ Each participant underwent measurement of anthropometric parameters, such as weight and standing height, by trained personnel. Height was measured to the nearest 0.1 cm with a stadiometer. Weight was measured to the nearest 0.5 kg with a balance scale. BMI was calculated as weight divided by the square of height (kg/m^2^) and used as an indicator of generalized obesity. Participants were categorized as having normal weight (BMI <25 kg/m^2^), being overweight (BMI: 25–29.9 kg/m^2^), and obese (BMI ≥30 kg/m^2^). Waist circumference (WC) was measured to the nearest 0.1 cm with a measuring tape at the level of the umbilicus and used as an indicator of central obesity. Participants were classified as having central obesity if WC was >102 cm in men or >88 cm in women. Four hundred sixty-six were considered in this study since they had complete demographic, anthropometric and health status information.

### Serum sampling and biochemical assays

Venus blood samples from patients was collected after overnight fasting and centrifuged at 3,000 rpm for 10 min. Serum was aliquoted and stored at −80°C until analysis. All the assays were performed by UV-VIS spectrophotometric techniques in a 96-well plate format by using a Tecan Infinite M200 microplate reader (Tecan group, Ltd., Maennedorf, Switzerland). The arylesterase activity was measured by adding 10 µl of serum to 240 µl of reaction mixture consisting in 1 mmol/L phenylacetate and 0.9 mmol/L CaCl_2_ dissolved in 9 mmol/L Tris-HCl, pH = 8.0.^(20)^ A molar extinction coefficient of 1.3 × 10^3^ (L/mol·cm) was used for the calculation of enzyme activity, expressed in kilo unit per liter. One unit of arylesterase activity accounts for 1 µmol of phenol produced in a minute under the conditions of the assay. The intra-assay CV was 3.8% whereas the inter-assay CV was 9.7%.

PON1 lactonase activity was measured using gamma-thiobutyrolactone (TBL) as substrate and Ellman’s procedure was used to spectrophotometrically monitor (412 nm) the accumulation of free sulfhydryl groups via coupling with 5,5-dithiobis(2-nitrobenzoic acid) (DTNB).^(23)^ The reaction was started by adding 10 µl of sample to 190 µl of mix containing buffer (50 mmol/L Tris, 1 mmol/L CaCl_2_, 50 mmol/L NaCl, pH = 8.0), 50 mmol/L DTNB and 10.5 mmol/L TBL in each well. A molar extinction coefficient of 13.6 × 10^3^ (L/mol·cm) was used for the calculation of enzyme activity, expressed in unit per liter. The intra-assay CV was 6.1% whereas the inter-assay CV was 9.8%. Total cholesterol (Tc), HDL-c, triglycerides and glucose were assayed by standard enzymatic-colorimetric methods and LDL-c was calculated according to the Friedewald formula.

### Statistical analysis

Continuous variables were first analyzed for normal distribution using Kolmogorov-Smirnov and Shapiro-Wilk tests. Group comparisons were performed using *t* test and Mann-Whitney *U* test for normally and non-normally distributed variables, respectively. Chi-squared test was used to compare categorical variables between groups. Simple correlation analyses were performed using Pearson’s and Spearman’s tests for normally and non-normally distributed variables, respectively. Since the distribution of some variables of interest (such as lactonase, arylesterase, BMI, etc.) became normal upon base-10 logarithm transformation, we used the log values for correlation analyses and subsequent multivariate test (to satisfy the fundamental assumption of multivariate analysis). Stepwise multiple regression analysis was used to determine the independence of the found associations involving arylesterase or lactonase. In this test, the aforementioned log-transformed variables were Criteria for variable inclusion in the stepwise regression analysis were entry if *p*≤0.05 and exclusion if *p*>0.10. Preliminary multiple regression analyses were performed to assess multicollinearity among variables included in the multivariate analyses. Values of variance inflation factor (VIF) above 2.5 were considered indicative of the presence of this statistical problem (waist circumference and BMI were not included in the same model because of their collinearity). Multivariate logistic regression analysis was performed to evaluate the association between high levels of arylesterase/lactonase and the risk of overall or abdominal obesity. A *p*<0.05 was considered statistically significant.

## Results

### Influence of sex on PON1 activities

The principal demographic, anthropometric and laboratory (lipid profile) characteristics of the men and women enrolled in our study are summarized in Table 1. The two sample groups did not differ for age, BMI and prevalence of overall obesity, whereas they did differ for waist circumference and abdominal obesity frequency (higher in women). Regarding lipids, the only significant difference was detected for HDL-c and total cholesterol, which were both higher in women compared to men.

**Fig. 1:**
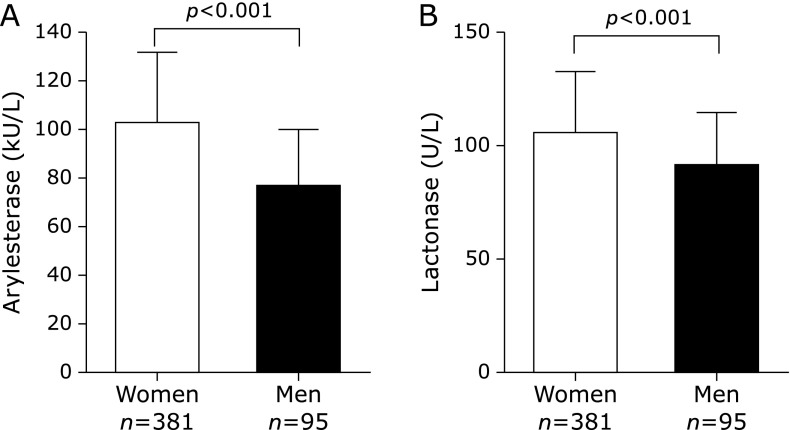
Arylesterase (A) and lactonase (B) activities measured in the serum of women and men. As depicted in the panels, both activities of PON1 were significantly higher in women than men (*p*<0.001 for both comparisons). The bars in the graph represent the mean ± SE.

As shown in Fig. 1, both arylesterase (Fig. 1A) and lactonase (Fig. 1B) activities of PON1 were significantly higher in women compared to men, with a difference between the means of 25 and 13%, respectively (see also Supplemental Table 1*). Since HDL is the main PON1 carrier, and the activity of PON1 relates with the level of this lipoprotein,^(8)^ we checked if the difference in the activities remained unchanged when the concentration of HDL-c were similar in men and women. To this end, a subsample of 105 women and 65 men with HDL-c below 50 mg/dl was randomly selected (women, HDL-c, mean ± SD, 41 ± 8 mg/dl; men, 39 ± 9 mg/dl) (Supplemental Fig. 1*). Within this subset, both arylesterase and lactonase activities were significantly higher in women (by 24 and 14%, respectively), thus confirming the data on the whole sample. Of note, the significance level of difference found at univariate analysis remained almost unaltered after controlling for age, smoking status and anthropometric variables.

Since it has been suggested that estrogens may influence serum PON1 activity in women, and that menopause affects PON1,^(24–26)^ we examined the sex difference in arylesterase/lactonase activities within two age intervals: 1) <45 years, corresponding (with approximation) to the female reproductive stage (high E2); 2) >55 years, corresponding to the postmenopausal stage (low E2).

We found that the distinction between men and women was markedly larger in the older compared to the younger subsample, with the difference between the means increasing from 18 to 32% for arylesterase, and from 8 to 19%, for lactonase (Supplemental Table 2*).

Association between PON1 activities and anthropometric parameters in women and men. Arylesterase was (although weakly) inversely correlated with both BMI (*r* = −0.181, *p*<0.001) and waist circumference (*r* = −0.248, *p*<0.001) in the whole sample (Supplemental Fig. 2A and B*, respectively). Stepwise multiple regression analysis revealed that these two univariate associations were strongly confounded by covariates such as age, sex, smoking and HDL-c (Supplemental Table 3*). Indeed, the correlation with BMI was much weaker (β = −0.095, *p* = 0.03) while that with waist circumference was no longer significant. Regarding lactonase, the association was absent with BMI and very weak with waist circumference (*r* = −0.123, *p* = 0.04) (Supplemental Fig. 2C and D*). Of note, the only significant association emerged for lactonase disappeared upon adjusting for age and sex (data not shown).

**Fig. 2:**
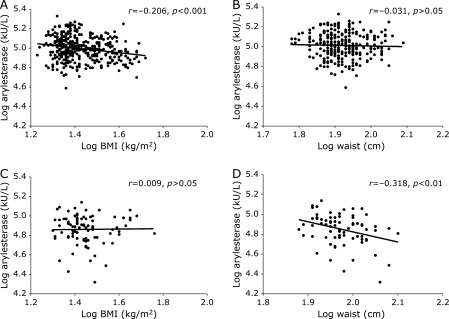
Correlation of arylesterase activity with body mass index (BMI) and waist circumference for women (A, B) and men (C, D). Arylesterase activity was significantly negatively correlated with BMI only in women, whereas it was negatively correlated with waist circumference only in men. All the values are expressed as Log_10_.

To evaluate the possible effect of sex on the interplay between PON1 activities and anthropometric measurements, we crossed these variables separately in women and men. From these analyses it emerged that arylesterase was significantly correlated with BMI in women (Fig. 2A; *r* = −0.248, *p*<0.001) but not in men (Fig. 2B; *r* = −0.031); conversely, this activity was correlated with waist circumference in men (Fig. 2D; *r* = −0.318, *p* = 0.005) but not in women (Fig. 2C; *r* = 0.009). As for arylesterase, also lactonase showed a significant association with waist circumference in men (Supplemental Fig. 3D; *r* = −0.275, *p* = 0.016), while no other correlations involving lactonase and anthropometric indexes reached a significance level (Supplemental Fig. 3*). As already observed in the total sample, the adjustment for potential confounders (age, HDL-c and smoking) affected the strengths of the found correlations; indeed, only arylesterase vs BMI in women remained significant at multivariate analysis (β = −0.149, *p* = 0.01).

Notably, the aforementioned correlation coefficients obtained with Log10-trasformed variables were highly similar to those emerged from analysis with non-transformed variables (Supplemental Fig. 4–6*).

Thus, the only sex-dependent relationship emerging from our analysis was the one between arylesterase and BMI. In the attempt to translate this result in a more practical/clinical setting, we checked whether high levels of arylesterase (levels above the median) were associated with a lower risk of being affected by overall obesity (BMI >30 kg/m^2^) in either of the two subsamples (men and women). In a way consistent with our previous findings, the multivariate logistic analysis showed that having high level of this PON1 activity was related with a lower probability of obesity (odds ratio, OR: 0.559 95% confidence interval, CI—95%: 0.340–0.919) in women, but not in men (OR 0.662; CI— −95%: 0.231–1.893).

### The interplay between arylesterase and BMI across age intervals

**Fig. 3:**
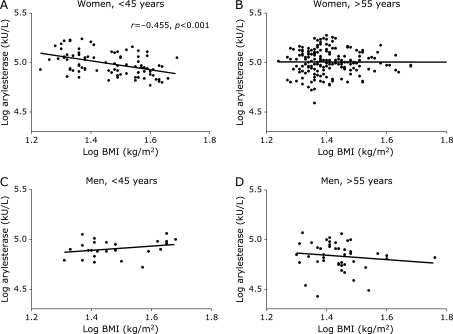
Correlation of arylesterase activity with body mass index (BMI) in women and men younger (A, C) and older (B, D) than 55 years. Arylesterase activity was negatively correlated with BMI in women younger than 45 years but not in those older than 55 years. On the contrary, men did not show any significant correlation between the two variables. All the values are expressed as Log_10_.

To search for useful clues to interpret our results, we analyzed how the interaction between arylesterase and BMI “fluctuated” across the two age intervals (1) <45 years and 2) >55 years) that, as already commented, approximately correspond to two distinct phases of women reproductive stages. We found interesting to perform this analysis because these two phases are frequently associated with a distinct body fat distribution: 1) normo-menstruating women exhibit a gynoid fat, characterized by a peripheral, gluteo-femoral, distribution;^(6)^ 2) postmenopausal women present a typical male- pattern, with a preferential accumulation of fat in the central (abdominal-visceral) depot.^(6)^ Age-cut-offs were chosen on the basis of the most recent data on epidemiology of menopause in Italy.^(27)^ As depicted in Fig. 3 (Supplemental Fig. 7* with non-log data), arylesterase and BMI did correlate in the “younger” women (Fig. 3A, *r* = −0.455, *p*<0.001) but not in older women (Fig. 3B, *r* = −0.002, *p*>0.05), and in either of the two corresponding men subsamples (Fig. 3C and D). Notably, the strength of the found association remained almost unaltered after controlling for potential confounders, including HDL-c (data not shown).

## Discussion

Potential confounding variables have to be taken into account in the design and analysis of epidemiological studies. In the case of PON1, little is known about the biological/environmental factors that impact its expression and activity level. Here we showed that sex not only might affect arylesterase and lactonase activities of PON1, but also it affects their relationship with clinical measures of obesity, such as BMI and waist circumference.

Both arylesterase and lactonase were increased in women compared to men, and this difference was independent of age, HDL-c and smoking. This result was consistent with the majority of studies on animals (within the same species and strain).^(28,29)^ The effect of mixture of progesterone/E2 on female animals and *in vitro* experiments on human and rat hepatic cells suggest that PON1 might be upregulated by E2.^(30,31)^ As highlighted elsewhere,^(32)^ sex differences in humans would be expected to be minimized by the genetic diversity of the population. As a proof of concept, not all epidemiological/clinical studies found significant difference in PON1 activities between men and women.^(25,32–34)^ Moreover, studies dealing with the effect of menopause and hormone replacement therapy on PON1 yielded conflictual results.^(25,26,35,36)^ In our study, menopause does not seem to correlate with a significant decrease in PON1 activity; in fact, women over 55 years of age had comparable levels of arylesterase and lactonase activity than those under 45 years.

Modification of body fat distribution, characterized by a rapid shift from “healthy” subcutaneous to “unhealthy” visceral sites, is also typically associated with menopause.^(4,37,38)^ Obesity and visceral obesity have been found to be inversely associated with PON1 activities in some,^(39–41)^ but not all,^(8,17,42)^ studies on general population. These discrepancies are more likely the result of differences in the composition and size of population sample, study design, and in analytical procedures employed to assay PON1 (several studies only measured paraoxonase activity). In our total population, only the inverse correlation between arylesterase and BMI was independent from the considered confounding factors. However, this association became weaker after full-adjustment, and this was mainly due to the strong influence of HDL-c and sex on PON1. Consistently, arylesterase was significantly correlated with BMI (and overall obesity prevalence) in women but not in men, and this association was significant only in women younger than 45 years.

It is well-established that gluteo-femoral adipose tissue relative contribution in total fat mass is greater in women than in men during lifetime, but mostly at younger (reproductive) age. This consideration and the limited/absent contribution of central fat in PON1 activity have led us to hypothesize that gluteal-femoral fat could be determinant for the inverse relationship between arylesterase and BMI.

This apparent negative consequence of peripheral adiposity on PON1 and on athero-protective function of HDL may not be reflected in a net worsening of metabolic profile; indeed, it might be counterbalanced by the well-known beneficial effects of peripheral subcutaneous fat, such as improved insulin sensitivity and a lower risk of developing type 2 diabetes, dyslipidemia and atherosclerosis.^(43)^ The most likely mechanism for metabolic differences between central and peripheral obesity relates to adipokines.^(43)^ This plethora of bioactive peptides or proteins, immune molecules, and inflammatory mediators are selectively (and in a sex-dependent manner) secreted by visceral, abdominal or gluteo-femoral subcutaneous fat.^(37,44)^ Future studies might be addressed to investigate whether the adipokines predominantly produced by gluteo-femoral adipose tissue,^(45)^ such as leptin and adiponectin, are implicated in the regulation of PON1 expression.

Finally, some important limitations and strengths of the study must be emphasized. First, the cross-sectional design precluded the ability to determine with certainty any cause-and-effect relationships between the measured variables. Thus, any statements about the downstream/upstream position of a factor with respect to another are hypothetical. Second, subclinical or undetected diseases, and potential confounding factors not considered in the study (e.g., diet, metabolic syndrome), and the unequal size of sample groups might have affected the reliability of the results. Third, anthropometric measures, although being by far the most used in epidemiological studies, are not the most accurate indexes of total adiposity and fat distribution.

In spite of these undeniable caveats, this explorative study has the strength to provide the rationale of a more comprehensive investigation on biological factors that can influence the activity of unconventional CVD risk factor as PON1. The future studies should be of longitudinal nature and should include the measurements of adipokines and body fat distribution using more rigorous methodologies (e.g., dual-energy X-ray absorptiometry computed tomography DEXA or tomography) able to distinguish subcutaneous from visceral fat in age- and sex-matched samples.

In conclusion, our study demonstrated a sex difference in PON1 activities, with women exhibiting higher levels of both serum lactonase and arylesterase activities compared to men. Sex dual dimorphism was also observed in the relationship of total and central fat, as assessed by BMI and waist circumference, with PON1 level. Specifically, we found that in women, but not in men, arylesterase activity was independently related with BMI. At a more in depth look at the data it emerged that this association was exclusive of the younger women (<45 years), thus suggesting a role of peripheral (gluteo-femoral) fat in influencing PON1 activity.

## Figures and Tables

**Table 1:** Main characteristics of women/men enrolled in the study

	Women (*n* = 374)	Men (*n* = 92)	*p*
Age (years)	52 ± 10	54 ± 12	>0.05
Smoking (%)	25	29	>0.05
Anthropometric parameters			
BMI (kg/m^2^)	28 ± 7	29 ± 7	>0.05
Overall obesity (%)	29	30	>0.05
Waist circumference (cm)	84 ± 10	95 ± 11	<0.001
Abdominal obesity (%)	63	50	<0.05
Lipid profile			
HDL-c (mg/dl)	61 ± 20	45 ± 13	<0.001
LDL-c (mg/dl)	141 ± 47	123 ± 46	>0.05
Triglycerides (mg/dl)	97 ± 73	101 ± 50	>0.05
Total cholesterol (mg/dl)	220 ± 52	196 ± 51	<0.001

Data presented are expressed as % within the group for categorical variables; mean ± SD for continuous variables. BMI, body mass index; HDL-c, high density lipoprotein cholesterol; LDL-c, low density lipoprotein cholesterol.
