# Supplementary material for: Sex difference: an important issue to consider in epidemiological and clinical studies dealing with serum paraoxonase-1
Source: J Clin Biochem Nutr. 2019 Jan 30;64(3):250–6. doi: 10.3164/jcbn.18-73 (PMC6529704; doi:10.3164/jcbn.18-73)
Supplement: Supplemental Figure 1 [file jcbn18-73sf01.pdf]

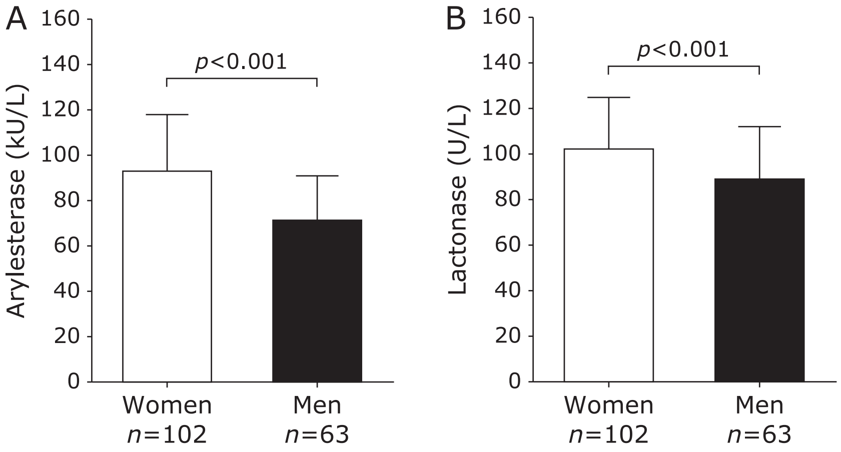

Supplemental Fig. 1. Arylesterase (A) and lactonase (B) activities measured in a subsample of women and men with a similar concentration of HDL cholesterol (below 50 mg/dl). Within this subset, both PON1 activities were higher in women than in men ($p<0.001$). The bars represent the mean ± SE.
