# Supplementary material for: Sex difference: an important issue to consider in epidemiological and clinical studies dealing with serum paraoxonase-1
Source: J Clin Biochem Nutr. 2019 Jan 30;64(3):250–6. doi: 10.3164/jcbn.18-73 (PMC6529704; doi:10.3164/jcbn.18-73)
Supplement: Supplemental Figure 2 [file jcbn18-73sf02.pdf]

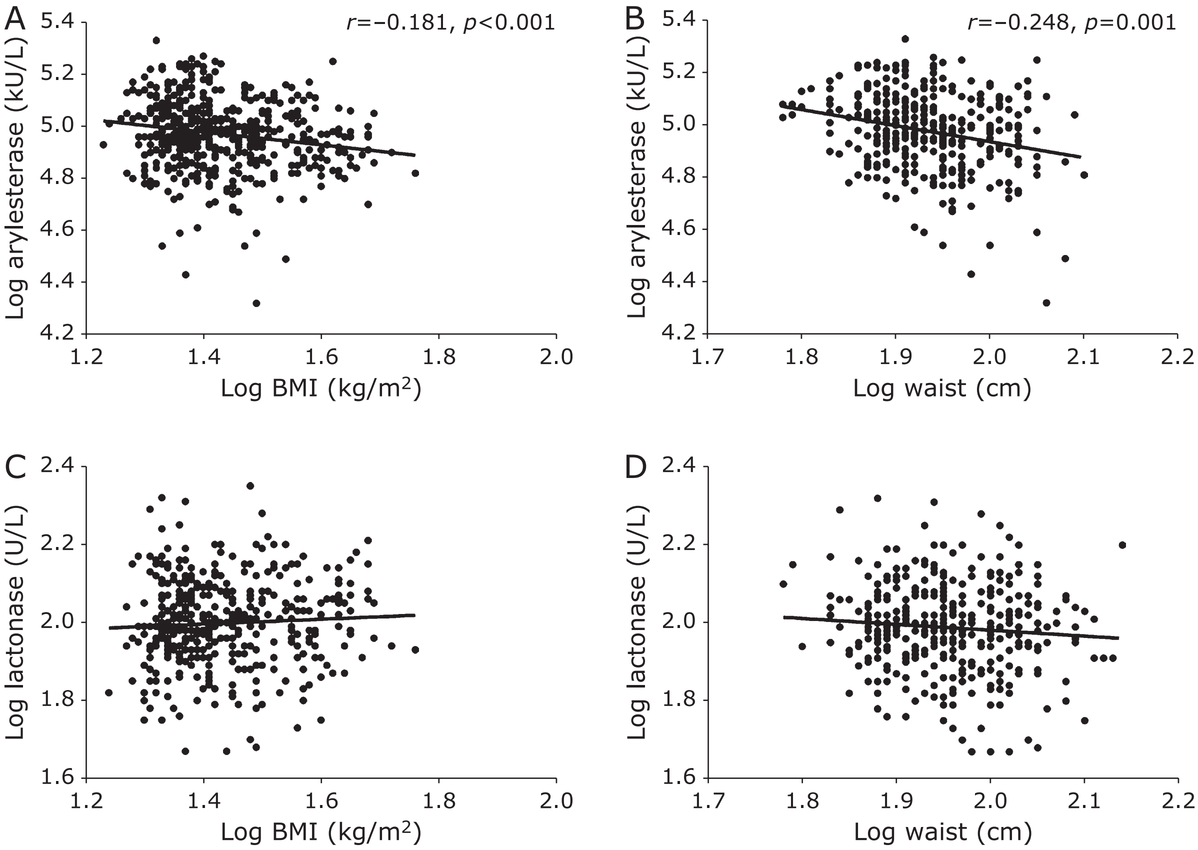

Supplemental Fig. 2. Correlation of arylesterase and lactonase activities with body mass index (BMI) and waist circumference in the whole population. Arylesterase was significantly and negatively correlated with BMI (A, r = –0.181, p<0.001) and waist circumference (B, r = –0.248, p<0.001) in the whole population. On the contrary, lactonase was not related with BMI (C) and showed only a very weak negative association with waist circumference (D, r = –0.123, p = 0.04). All the values are expressed as Log_{10}.
