# Supplementary material for: Sex difference: an important issue to consider in epidemiological and clinical studies dealing with serum paraoxonase-1
Source: J Clin Biochem Nutr. 2019 Jan 30;64(3):250–6. doi: 10.3164/jcbn.18-73 (PMC6529704; doi:10.3164/jcbn.18-73)
Supplement: Supplemental Figure 4 [file jcbn18-73sf04.pdf]

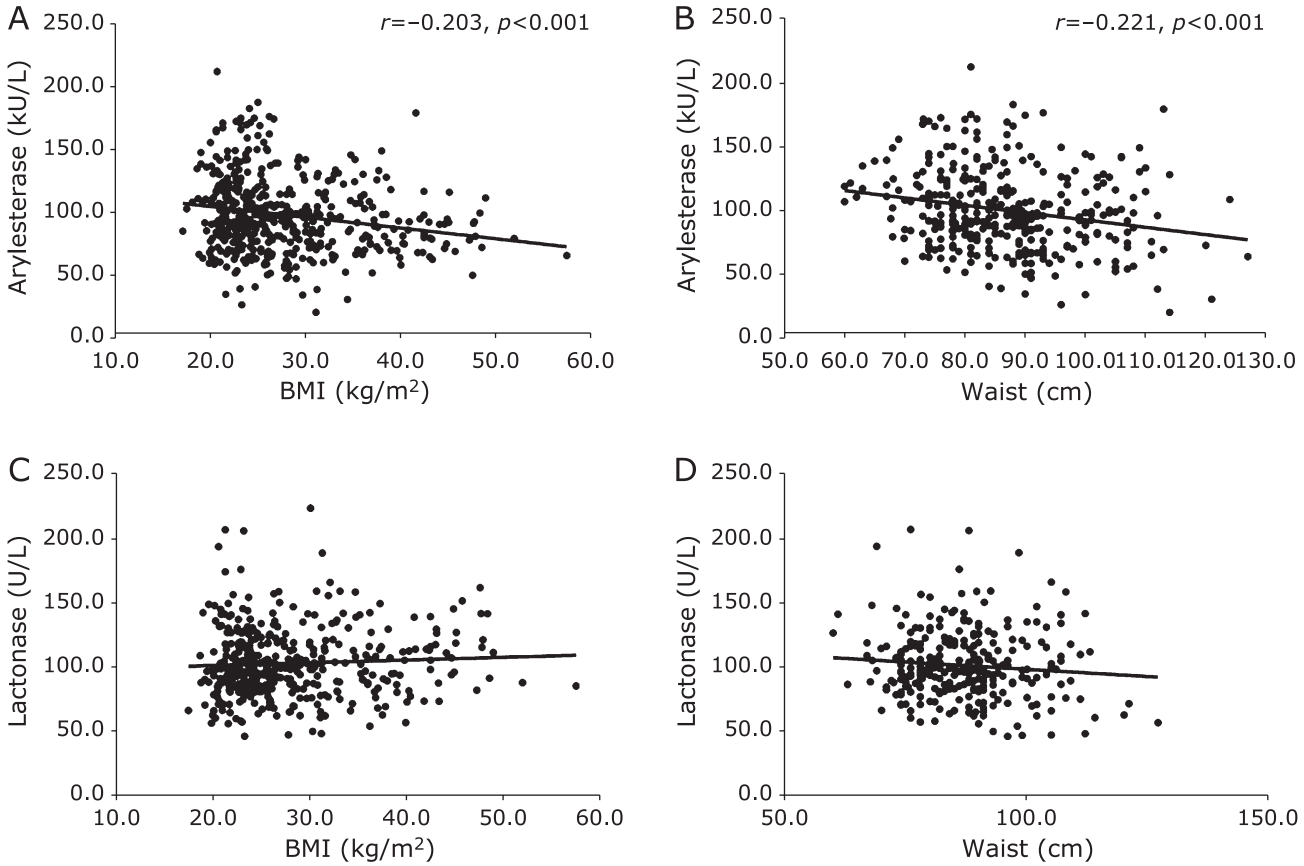

Supplemental Fig. 4. Correlation of arylesterase (A, B) and lactonase (C, D) activities with body mass index (BMI) and waist circumference in the whole population. Arylesterase was significantly and negatively correlated with BMI (A, r = –0.203, p<0.001) and waist circumference (B, r = –0.221, p<0.001) in the whole population. On the contrary, lactonase was not related with either BMI (C) or waist circumference (D). These scatter plots display the same data of Supplemental Fig. 2 before logarithmic transformation.
