# Supplementary material for: Sex difference: an important issue to consider in epidemiological and clinical studies dealing with serum paraoxonase-1
Source: J Clin Biochem Nutr. 2019 Jan 30;64(3):250–6. doi: 10.3164/jcbn.18-73 (PMC6529704; doi:10.3164/jcbn.18-73)
Supplement: Supplemental Figure 5 [file jcbn18-73sf05.pdf]

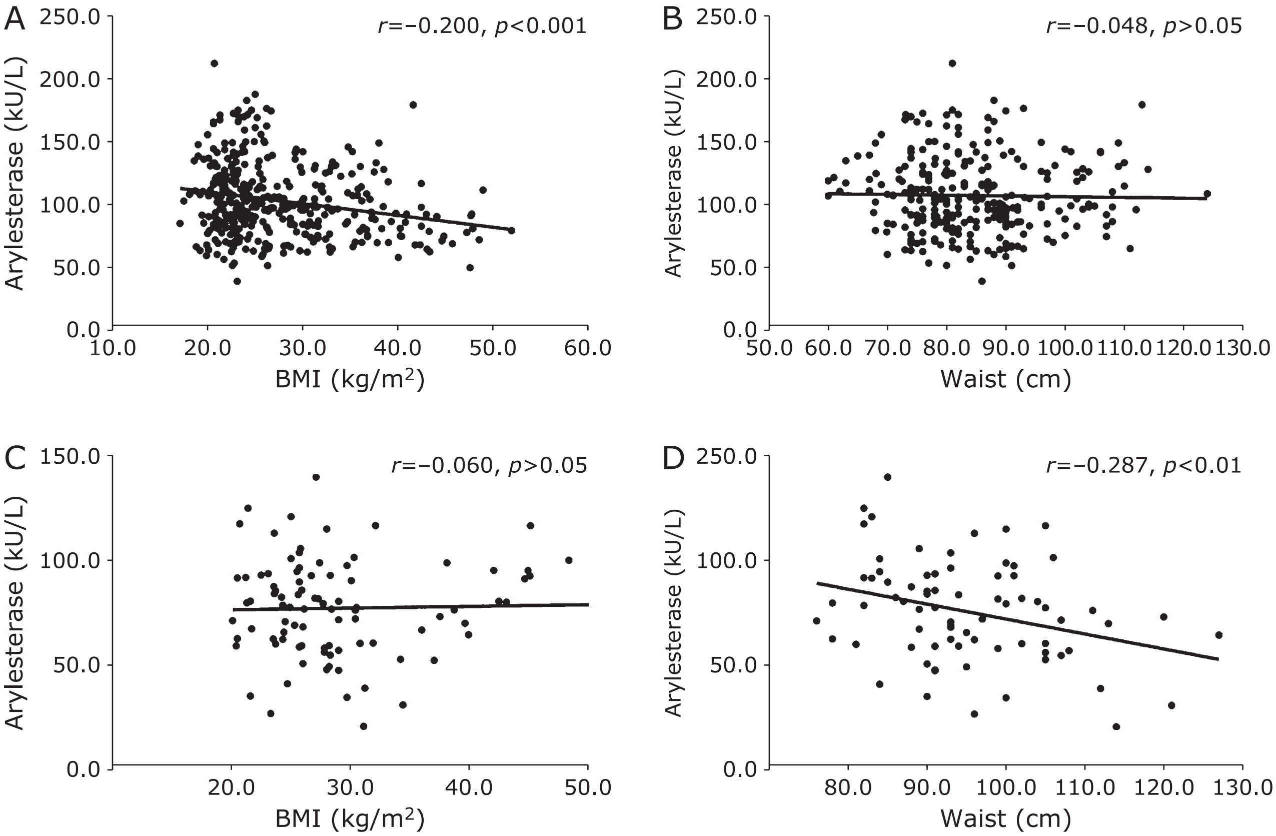

Supplemental Fig. 5. Correlation of arylesterase activity with body mass index (BMI) and waist circumference for women (A, B) and men (C, D). Arylesterase activity was negatively correlated with BMI in women ($r = -0.200$, $p<0.001$) (A), but not in men (C). The activity was inversely associated with waist circumference in men ($r = -0.287$, $p<0.01$) (D), but not in women (B). These scatter plots display the same data of Fig. 2 before logarithmic transformation.
