# Supplementary material for: Sex difference: an important issue to consider in epidemiological and clinical studies dealing with serum paraoxonase-1
Source: J Clin Biochem Nutr. 2019 Jan 30;64(3):250–6. doi: 10.3164/jcbn.18-73 (PMC6529704; doi:10.3164/jcbn.18-73)
Supplement: Supplemental Figure 6 [file jcbn18-73sf06.pdf]

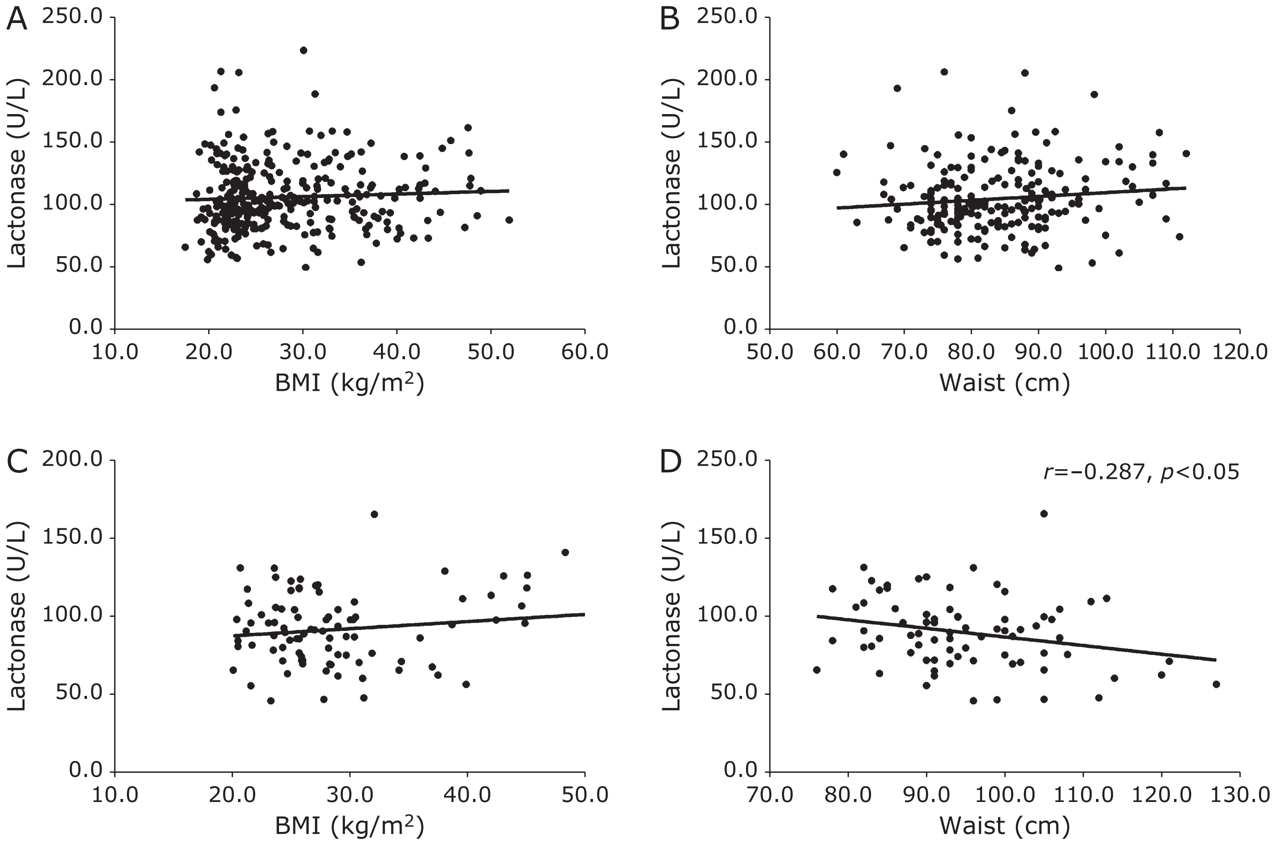

Supplemental Fig. 6. Correlation of lactonase activity with body mass index (BMI) and waist circumference in women (A, B) and men (C, D). Lactonase was not correlated with BMI (A) or waist circumference (B) in women. In men, lactonase was not related with BMI (C) but showed a significant negative relation with waist circumference (D, $r = -0.287$, $p<0.05$). These scatter plots display the same data of Supplemental Fig. 3 before logarithmic transformation.
