# Supplementary material for: Sex difference: an important issue to consider in epidemiological and clinical studies dealing with serum paraoxonase-1
Source: J Clin Biochem Nutr. 2019 Jan 30;64(3):250–6. doi: 10.3164/jcbn.18-73 (PMC6529704; doi:10.3164/jcbn.18-73)
Supplement: Supplemental Figure 7 [file jcbn18-73sf07.pdf]

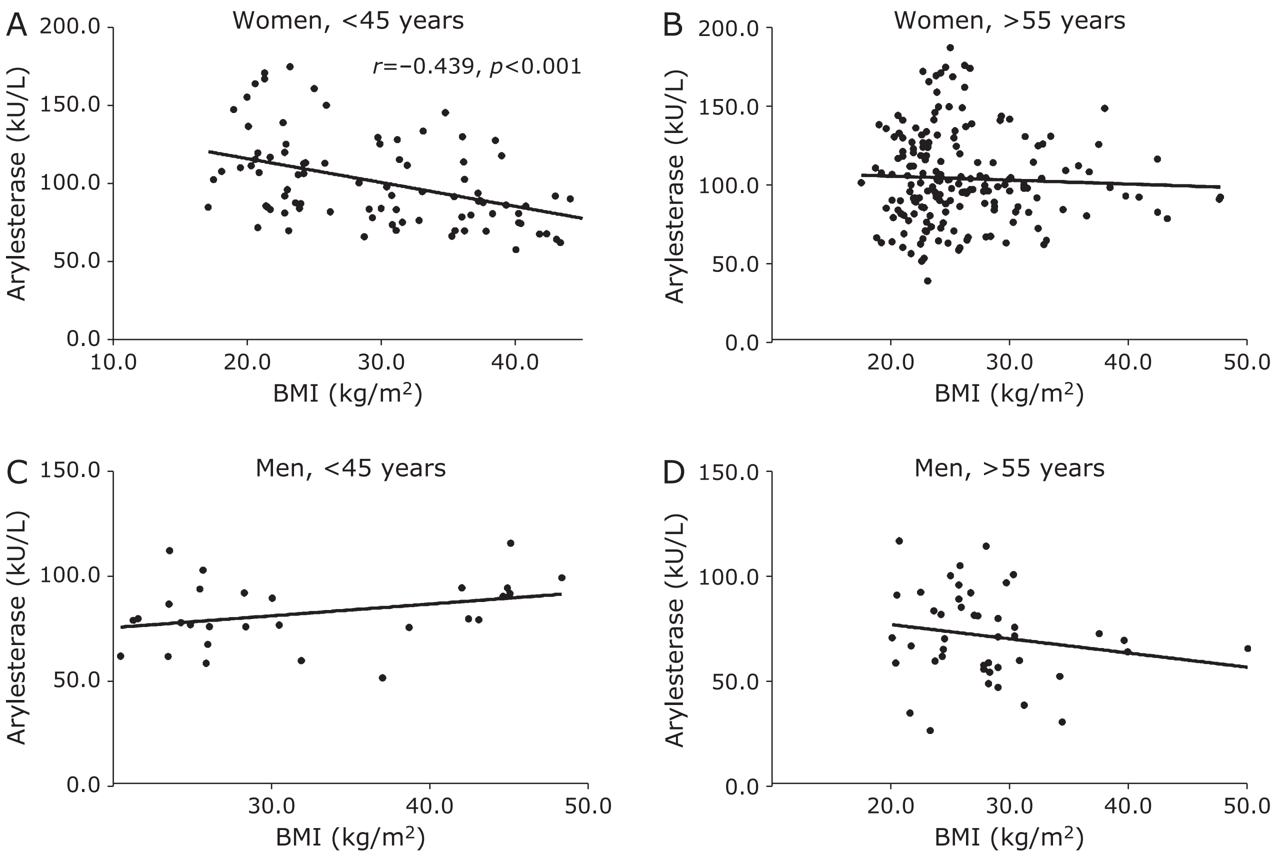

Supplemental Fig. 7. Correlation of arylesterase activity with body mass index (BMI) in women and men younger (A, C) and older (B, D) than 55 years. Arylesterase activity was negatively correlated with BMI ($r = -0.439$, $p<0.001$) in women younger than 45 years (A) but not in the older ones (B). On the contrary, men did not show any correlations between the two variables (C, D). These scatter plots display the same data of Fig. 3 before logarithmic transformation.
