# Supplementary material for: Sex difference: an important issue to consider in epidemiological and clinical studies dealing with serum paraoxonase-1
Source: J Clin Biochem Nutr. 2019 Jan 30;64(3):250–6. doi: 10.3164/jcbn.18-73 (PMC6529704; doi:10.3164/jcbn.18-73)
Supplement: Supplemental Table 1 [file jcbn18-73st01.pdf]

Supplemental Table 1. Serum Arylesterase/Lactonase activities of PON1 in women and men

	Women (n = 374)	Men (n = 92)	p
PON1 activities			
Arylesterase (kU/L)	103 ± 29	77 ± 22	<0.001
Lactonase (U/L)	106 ± 27	92 ± 22	<0.001

Data presented are expressed as mean ± SD.
