# Supplementary material for: Sex difference: an important issue to consider in epidemiological and clinical studies dealing with serum paraoxonase-1
Source: J Clin Biochem Nutr. 2019 Jan 30;64(3):250–6. doi: 10.3164/jcbn.18-73 (PMC6529704; doi:10.3164/jcbn.18-73)
Supplement: Supplemental Table 2 [file jcbn18-73st02.pdf]

Supplemental Table 2. Serum Arylesterase/Lactonase activities of PON1 in women and men aged <45 or >55 years

	Age <45 years			Age >55 years		
	Women (*n* = 88)	Men (*n* = 31)	Difference men/women (%)	Women (*n* = 88)	Men (*n* = 31)	Difference men/women (%)
PON1 Activities						
Arylesterase (kU/L)	101 ± 28	83 ± 16[a]	18	104 ± 29	72 ± 21[a]	31
Lactonase (U/L)	105 ± 26	97 ± 23	8	106 ± 26	86 ± 22[a]	19

Data presented are expressed as mean ± SD. [a]$p<0.001$ vs Women.
