# Supplementary material for: Sex difference: an important issue to consider in epidemiological and clinical studies dealing with serum paraoxonase-1
Source: J Clin Biochem Nutr. 2019 Jan 30;64(3):250–6. doi: 10.3164/jcbn.18-73 (PMC6529704; doi:10.3164/jcbn.18-73)
Supplement: Supplemental Table 3 [file jcbn18-73st03.pdf]

Supplemental Table 3. Stepwise multiple regression analysis of the contribution of BMI or waist circumference on arylesterase variance (n = 466)

Regression models*	Parameter estimate (SE)	Standardized coefficient	p	Overall model R^2 (p value)
1				
Log_{10} BMI	–0.242 (0.061)	–0.181	>0.001	3.3 (<0.001)
2				
Gender	–0.107 (0.015)	–0.312	>0.001	
HDL-c	–0.002 (0.000)	–0.221	>0.001	
Log_{10} BMI	–0.126 (0.056)	–0.095	>0.05	21.6 (<0.001)
3				
Log_{10} waist	–0.616 (0.128)	–0.248	>0.001	6.1 (<0.001)
4				
Gender	–0.131 (0.018)	–0.382	>0.001	
HDL-c	–0.002 (0.000)	–0.222	>0.001	25.7 (<0.001)

Regression models 1 and 3 are univariate models with Log_{10} BMI (1) or Log_{10} waist (3) as explanatory variables. Multiple regression models 2 and 4 included the following covariates: age, gender, smoking and Log_{10} BMI (2) or Log_{10} waist (4). Criteria for variable inclusion in the stepwise regression analysis were entry if $p \leq 0.05$ and exclusion if $p > 0.10$. BMI, Body Mass Index; HDL-c, high density lipoprotein cholesterol.
